# A Novel Method to Identify Three Quality Grades of Herbal Medicine Ophiopogonis Radix by Microscopic Quantification

**DOI:** 10.3389/fphar.2020.591310

**Published:** 2021-01-29

**Authors:** Kunzi Yu, Wei Liu, Nanping Zhang, Xianlong Cheng, Shiyu Zhou, Tiantian Zuo, Shuai Kang, Feng Wei, Shuangcheng Ma

**Affiliations:** ^1^ Institute for Control of Chinese Traditional Medicine and Ethnic Medicine, National Institutes for Food and Drug Control, Beijing, China; ^2^ Chengdu Institute for Food and Drug Control, Chengdu, China

**Keywords:** light microscope microscopy, herbal medicine, botanical identification, quality identification, microscopic quantification, ophiopogonis radix

## Abstract

Maidong, the root tuber of *Ophiopogon japonicus* (Thunb.) Ker Gawl., is a commonly used herbal medicine in China. There are three quality grades of Maidong according to traditional opinion and modern research studies: superior quality (Zhe-Maidong), medium quality (Chuan-Maidong), and poorest quality (Chuan-Maidong with paclobutrazol, which is a kind of plant growth regulator). However, no efficient way to distinguish the three quality grades of Maidong exists; thus, the herbal markets and botanical pharmacies are flooded with Chuan-Maidong with paclobutrazol. To ensure the safety and quality of Maidong, a comparative microscopic study was performed on three quality grades of Maidong. The result was to establish a microscopic quantification method based on the area ratio between xylem and pith to distinguish the three quality grades of Maidong. Subsequently, Maidong from regional markets was evaluated by this method. In this study, we developed a novel quantification method to identify the three quality grades of Maidong, which could in turn make efforts on the quality improvement of Maidong. Our study is the first to demonstrate that microscopic technology could be used to distinguish different quality grades of a specific herbal medicine.

## Introduction

Maidong (Ophiopogonis Radix), which is the dried root tuber of *Ophiopogon japonicus* (Thunb.) Ker Gawl (family: Liliaceae) (the State Pharmacopoeia Committee of China, 2020), is one of the most important herbal medicines and has been used for over 2000 years in China. Maidong has been approved as a functional food ingredient (Lin et al., 2011) and as the main ingredient in patented drugs, such as ShenMai granule and XuanMai Gan Jie capsule/granule (the State Pharmacopoeia Committee of China, 2020). Recent studies have shown that Maidong mainly contains polysaccharides, steroidal saponins, and homoisoflavonoids (Nguyen et al., 2003; Chen et al., 2011; Zhang et al., 2012; Chen et al., 2016). Pharmacological research has revealed the anti-inflammatory, antioxidant, immunoregulatory, cardiovascular protective, and venous thrombosis inhibition effects of Maidong (Kou et al., 2005a; Kou et al., 2005b; Xiong et al., 2011; Zhang et al., 2016). Historically, the dried root tubers of *Liriope spicata* Lour. and L. *muscari* (Decne.) L. H. Bailey were used as Maidong, which resulted in nomenclatural confusion in herbal markets. In 2010, the microscopic characteristics have been described and helped distinguish authentic Maidong from the adulterant species (Mo, 2010). Therefore, distinguishing genuine or counterfeit Maidong is no longer an important issue.

The quality of Maidong is noteworthy and is influenced by at least two factors. The first influencing factor is the cultivation region. There are two main cultivation regions of Maidong in China: Zhejiang province and Sichuan province. Maidong cultivated in Zhejiang province is called Zhe-Maidong; that cultivated in Sichuan province is called Chuan-Maidong. Zhe-Maidong has been widely recognized as “daodi medicinal material,” a quality standard indicating that it has superior quality compared with Chuan-Maidong (Zhao et al., 2012; Li et al., 2016b). Modern research found that ophiopogonin B and ophiopogonin D contents in the tubers of Zhe-Maidong were higher than those in the tubers of Chuan-Maidong (Li et al., 2016a). Moreover, Zhe-Maidong showed higher promoting rates in macrophage phagocytosis and gastrointestinal motility than Chuan-Maidong, suggesting that the former has stronger immunomodulatory activities (Lu et al., 2017). The second influencing factor is the cultivation mode. According to our field survey, Zhe-Maidong needs 3-4 years for growth in the field, only a few of Chuan-Maidong is cultivated for 2 years, and most of Chuan-Maidong is cultivated for 1 year only and is overdosed with the plant growth regulator paclobutrazol to increase the yield two to three times. Based on a previous report, a flavonoid and four steroidal saponins were significantly decreased in Maidong after spraying paclobutrazol. In addition, different levels of paclobutrazol residue were detected in Maidong, soil and water samples, and the detection rate of paclobutrazol in Maidong was 100% (Zhang et al., 2019). The indiscriminate use of paclobutrazol leads to a growing concern about its safety.

When analyzing genetic differences between species, molecular biology techniques mainly rely on gene sequence comparison and calculation of conserved regions on chloroplasts and mitochondria. When analyzing genetic differences within species, biologists generally use gene expression profiles, but the long storage of herbal medicine makes it difficult to obtain plant gene expression profiles, which greatly limits the possibility of using genetic analysis to classify the quality grades of herbal medicine. Moreover, techniques based on chemical component analysis to identify the three quality grades have not been established, and thus no method to distinguish the different quality grades of Maidong exists. Based on this situation, Zhe-Maidong and Chuan-Maidong could barely be found in markets and Chuan-Maidong with paclobutrazol is rampant in the market, thereby reducing the medicinal quality of Maidong. In addition, the long planting cycle of Zhe-Maidong minimized the farmers’ economic interests; superior germplasm resource of Zhe-Maidong is reducing and vanishing gradually (Li et al., 2016b). Therefore, establishing a method to distinguish the three quality grades of Maidong is vital.

Microscopic technology has the advantages of speed, simplicity, reliability, requires small samples amounts, and is low cost. This method is applied in many pharmacopoeias (Zhao et al., 2006) and has been successfully used to identify herbal medicines and authenticate Chinese prescriptions (Liu et al., 2011; Kang et al., 2012; Xu et al., 2015). Moreover, histological techniques based on microscopic examination have been used to reveal the characteristics of tissue structure and arrangement of cells that could be used as markers for identifying original sources of plant-derived drugs, such as *Aloe vera* var. *chinensis* (Shen et al., 2001) and *Dendrobium officinale* Kimura et Migo (Yu et al., 2017). While microscopic and histological studies in quantitative analysis are limited, they are effective. For example, Wuzhimaotao (Radix Fici Hirtae) was identified by laticifer quantification (Au et al., 2009). However, distinguishing the same herbal medicine from different cultivation locations and with different cultivation modes based on microscopic techniques is not been developed yet, and the histological differences due to plant growth regulators have not been reported completely.

In this study, we established an analytical method to quantify microscopic characteristics; the quantification method was subsequently applied to examine Maidong from different cultivation regions and with different cultivation modes. This study aimed to provide scientific and objective data using microscopic quantification to identify the quality grades of Maidong, which could in turn promote the use of the best herbs and thus ensure the effectiveness and safety of Maidong.

## Materials and Methods

### Plant Material

Eighteen batches of Maidong, including four batches of Zhe-Maidong, four batches of Chuan-Maidong, and ten batches of Chuan-Maidong with paclobutrazol, from the field were used in this study. The details of each sample are presented in Table 1. To validate the method established by this study and evaluate the quality of Maidong in market, twelve batches of Maidong were obtained from herbal markets and botanical pharmacies in different provinces in China (Table 2). All samples were authenticated by Dr Shuai Kang (Institute for Control of Chinese Traditional Medicine and Ethnic Medicine, National Institutes for Food and Drug Control), and the voucher specimen (no. CH-056-100) was deposited in the National Institute for the Control of Pharmaceutical and Biological Products, National Institutes for Food and Drug Control, Beijing, China.

**TABLE 1:** Data of the three quality grades of Maidong.

Maidong	Source	Batch no.	Collection area	GPS coordinate	Collection date
Zhe-maidong	Field	Z1	Hangzhou, Zhejiang	E 118°753′, N 29°124′	March 2013
		Z2	Cixi, Zhejiang	E 121°279′, N 30°175′	September 2010
		Z3	Cixi, Zhejiang	E 122°563′, N 31°564′	May 2006
		Z4	Cixi, Zhejiang	E 121°751′, N 30°945′	August 2009
Chuan-maidong	Field	C1	Nanchong, Sichuan	E 105°367′, N 30°575′	April 2018
		C2	Nanchong, Sichuan	E 105°624′, N 30°863′	April 2018
		C3	Nanchong, Sichuan	E 106°157′, N 31°116′	April 2018
		C4	Nanchong, Sichuan	E 105°421′, N 31°254′	May 2018
Chuan-maidong with paclobutrazol	Field	CP1	Mianyang, Sichuan	E 104°561′, N 30°577′	November 2015
		CP2	Mianyang, Sichuan	E 105°241′, N 31°784′	August 2018
		CP3	Mianyang, Sichuan	E 103°875′, N 32°218′	August 2018
		CP4	Mianyang, Sichuan	E 105°116′, N 32°511′	August 2018
		CP5	Mianyang, Sichuan	E 104°335′, N 31°782′	August 2018
		CP6	Mianyang, Sichuan	E 104°951′, N 30°756′	April 2018
		CP7	Mianyang, Sichuan	E 105°223′, N 32°155′	April 2018
		CP8	Mianyang, Sichuan	E 104°596′, N 31°321′	April 2018
		CP9	Mianyang, Sichuan	E 105°332′, N 32°544′	April 2018
		CP10	Mianyang, Sichuan	E 103°651′, N 30°965′	May 2015

**TABLE 2:** Data of Maidong from the herbal markets and botanical pharmacies.

Batch no.	Collection area	Collection date
MS1	Chengdu, Sichuan; herbal market	May 2017
MS2	Hefei, Anhui; botanical pharmacies	May 2017
MS3	Shanghai; botanical pharmacies	May 2017
MS4	Mianyang, Sichuan; herbal market	May 2017
MS5	Wuhan, Hubei; botanical pharmacies	June 2017
MS6	Anguo, Hebei; herbal market	June 2017
MS7	Shijiazhuang, Hebei; botanical pharmacies	Nov 2020
MS8	Beijing; botanical pharmacies	Nov 2020
MS9	Bozhou, Anhui; herbal market	Nov 2020
MS10	Bozhou, Anhui; herbal market	Nov 2020
MS11	Guangzhou, Guangdong; herbal market	Nov 2020
MS12	Foshan, Guangdong; botanical pharmacies	Nov 2020

### Apparatus

All transverse sections of the materials were prepared using Leica Jung Biocut 2035 (Leica Instruments, Germany). A light microscope (Olympus BX51, Japan) equipped with an Olympus DP71 digital camera (Olympus, Tokyo, Japan) was used for image acquisition. Images were processed with a Zeiss AX10 equipped with a Zeiss AxioCam ICc five camera and analyzed by ZEN 2.3 lite (Zeiss, Germany). Area was measured in μm^2^.

Liquid chromatography/mass spectrometry (LC/MS) analysis was conducted using Agilent 1200 and Agilent 6410 Triple Quad LC/MS systems (Agilent Technologies, Santa Clara, CA, United States). A high-performance liquid chromatography (HPLC) analytical column (4.6 × 250 mm, 5.0 μm, ZORBAX SB-C18, Agilent, United States) was used at 40°C.

### Reagents

Chloral hydrate test solution was prepared using 50 g of chloral hydrate powder (Sinopharm Chemical Reagent, China), 15 ml of distilled water, and 10 ml of glycerinum (Sinopharm Chemical Reagent, China). Phloroglucinol test solution, which is a classic dye used to stain lignified cell walls red, was prepared using 0.5 g of phloroglucinol powder (Sinopharm Chemical Reagent, China) and 25 ml of 95% ethanol (Sinopharm Chemical Reagent, China). Diluted glycerin was prepared using 33 ml of glycerinum and 67 ml of distilled water. After the preparation, the aforementioned three solutions were filtered into dark-colored bottles and kept at room temperature. A paclobutrazol standard was purchased from Aladdin Industrial Corporation (P109932-250 mg, Shanghai, China); HPLC‐grade acetonitrile were purchased from Sinopharm Chemical Reagent. Pure water was prepared using a Milli-Q water purification system (Millipore, Burlington, MA, United States).

### Preparation of Standard and Market Sample Solution for LC/MS Analysis

The reference compound of paclobutrazol was weighed accurately and dissolved in acetonitrile to produce standard solutions. The samples from herbal markets and botanical pharmacies were powdered, and each powdered sample of 2 g (accurately weighed) was ultrasonicated with 20 ml of acetonitrile containing 0.1% formic acid for 30 min. Each solution was centrifuged at 3,000 rpm for 10 min. The supernatant was saved and filtered through a 0.45 μm filter for qualitative analysis.

## Method

**FIGURE 1:**
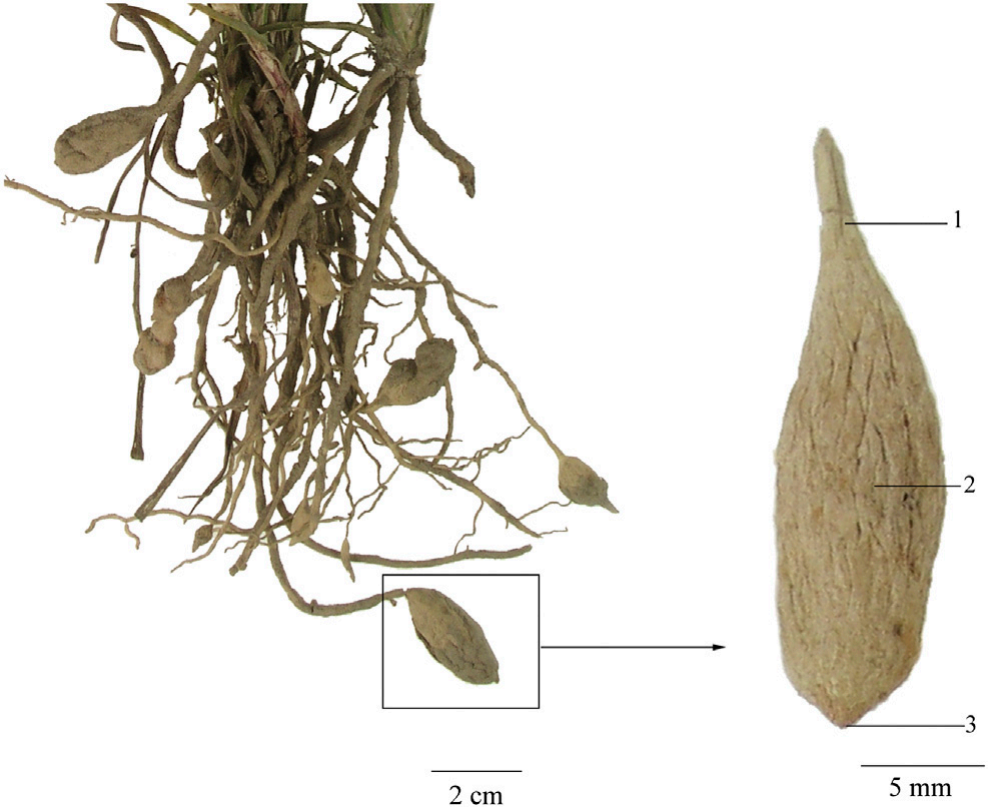
Sampling sites of Maidong: 1) upper site, 2) middle site, and 3) lower site.

For each batch of root tuber of *O. japonicus*, four samples were investigated. To investigate the microscopic characteristics of each sample in different section locations, tissues from the middle, upper, and lower sampling sites were sectioned and compared (Figure 1).

Samples were sectioned using Leica Jung Biocut 2035 to 30 μm thickness. Slides were prepared by adding one to two drops of chloral hydrate solution, rapidly heating to boiling repeatedly, and cleaning away excess chloral hydrate solution. One drop of phloroglucinol solution and hydrochloric acid stain, which was allowed to sit for 3 min, was used to stain lignified cells, and excess solution on the slide was washed away with diluted glycerin. Thereafter, the slides were sealed with diluted glycerin and observed under a microscope. The xylem and pith area of each sample was measured with ZEN 2.3 lite, and then data were analyzed using Statistical Package for the Social Sciences (SPSS).

The conditions for chromatographic separations were as follows: mobile phase consisted of water containing 0.1% formic acid and acetonitrile, and the isocratic elution program was 20% acetonitrile (20 min). The flow rate was 0.3 ml/min, and the injection volume was 5 μl. Mass spectra were detected in the positive mode. Moreover, the source parameters were as follows: dry gas (N_2_) temperature 350°C; flow rate 8 L/min; sheath gas flow 8 L/min with heater at 350°C; nebulizer pressure 45 psi; and capillary voltage 3500 V. The dwell time for each ion pair was 20 ms, and each sample was analyzed in triplicate.

## Results

### Testing and Identifying Field Samples

**FIGURE 2:**
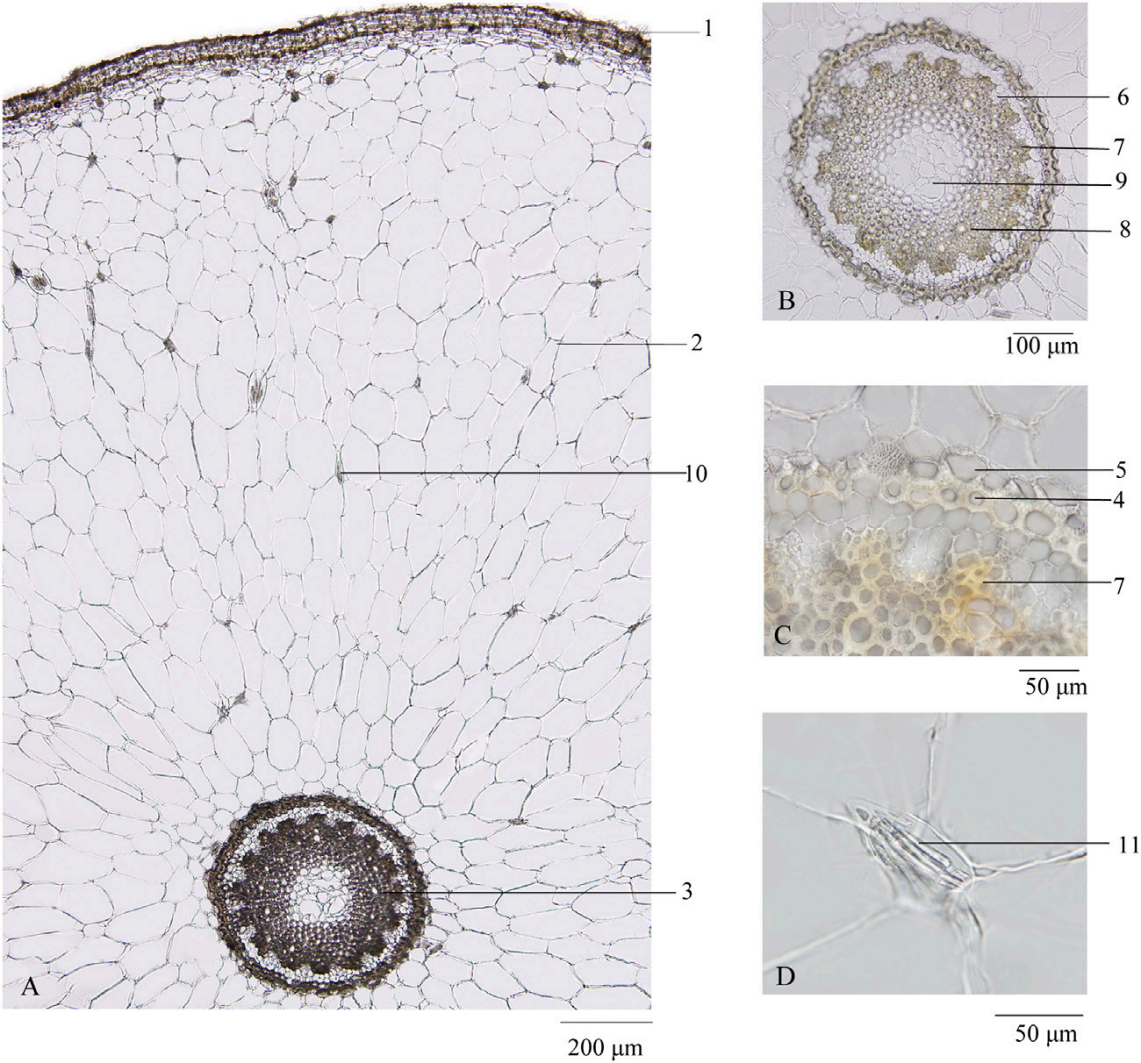
Transverse section of Maidong: **(A)** root transverse section; **(B)** stele; **(C)** endodermis; **(D)** thickened raphides of calcium oxalate. 1) Velamen, 2) cortex, 3) stele, 4) endodermis, 5) stone cells, 6) phloem, 7) protoxylem, 8) metaxylem, 9) pith, 10) raphides of calcium oxalate, and 11) thickened raphides of calcium oxalate.

Transverse section: Velamen consisting of 3–5 layers of lignified cells and cortex broad, showing scattered mucilage cells containing raphides of calcium oxalate, which seldom thickened to 5–10 μm in diameter; endodermal cells with evenly thickened and lignified walls, with subrounded cell cavity; and a layer of stone cells lying at the outside of endodermis, the inner and lateral walls thickened, and finely and densely pitted. Stele is relatively small, and 16–22 phloem bundles were noted. Protoxylem stellate and metaxylem linking up in a ring were observed. Pith cells were small, and parenchymatous cells were subrounded. According to Mo (Mo, 2010), thickened raphides of calcium oxalate, subrounded cell cavity of endodermal cells, and number of phloem bundles are the three main characteristics that distinguish genuine from adulterated Maidong (Figure 2).

The transverse sections of all the samples were investigated to ensure accuracy of the species.

### Investigation of Sampling Sites

To explore the influence of microscopic characteristics by sampling sites, the upper, middle, and lower sites were investigated separately (Figure 1).

Across all samples, the velamen and cortex were stable and showed no difference among the three quality grades of Maidong. Thus, this study focused on the microscopic characteristics of the endodermis and stele.

#### Zhe-Maidong

**FIGURE 3:**
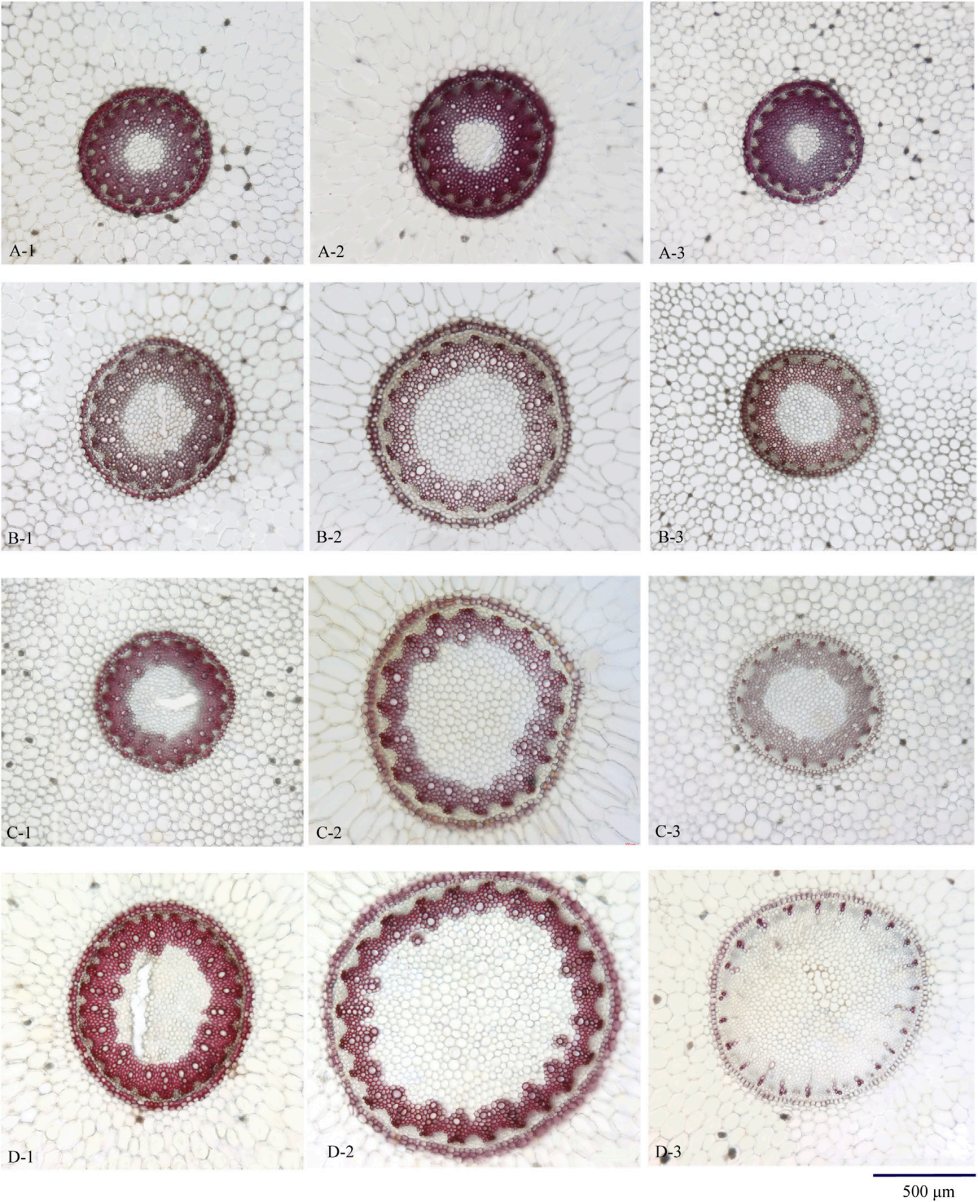
Transverse section of Maidong from different sampling sites: **(A)** Zhe-Maidong, **(B)** Chuan-Maidong, and **(C,D)** Chuan-Maidong with paelobutrazol. 1) Upper site, 2) middle site, and 3) lower site.

The diameter of stele and pith in the upper and lower sampling sites was smaller than that in the middle sampling sites. Microscopic elements including the thickness and lignification of endodermis and stone cells, the shape of protoxylem, and lignification of xylem showed no difference among the upper, lower, and middle sampling sites (Figure 3A, A-1, 2, 3).

#### Chuan-Maidong

The diameter of stele and pith in the upper and lower sampling sites was smaller than that in the middle sampling sites. The shape of the protoxylem in the upper and lower sampling sites was clearly distinct; the protoxylem in the upper sampling site was obtusely rounded and that in the lower sampling site showed acute angles. The thickness and lignification of endodermis and stone cells and the lignification of xylem showed no difference among the upper, lower, and middle sampling sites. In addition, the pith area was larger in Chuan-Maidong than in Zhe-Maidong, and the thickness and lignification of endodermis and stone cells were lower in Chuan-Maidong than in Zhe-Maidong (Figure 3B, B-1, 2, 3).

#### Chuan-Maidong With Paclobutrazol

The diameter of stele and pith in the upper and lower sampling sites was smaller than that in the middle sampling site. The shape of the protoxylem in the upper and lower sampling sites was clearly distinct. The protoxylem in the upper sampling site was obtusely rounded and that in the lower sampling site showed acute angles (Figure 3C, C-1, 2, 3). Furthermore, the metaxylem was undeveloped and could not link up to a ring in 48% (19 of 40) of the Chuan-Maidong with paclobutrazol (Figure 3D, D-3). The thickness and lignification of endodermis and stone cells and the lignification of xylem in the lower sampling site were much less than those in the upper and middle sampling sites. In addition, only a few stone cells with thickened and lignified cell walls were located outside the endodermis in middle and lower sampling sites in some of the Chuan-Maidong with paclobutrazol samples. The pith area was larger in Chuan-Maidong with paclobutrazol than in Zhe-Maidong, and the thickness and lignification of endodermis and stone cells in Chuan-Maidong with paclobutrazol were less than those of Zhe-Maidong (Figure 3).

### Microscopic Quantification

The microscopic characteristics of Maidong were influenced by the sampling sites in our study (see Investigation of Sampling Sites section). Thus, in the comparative study among the three quality grades of Maidong, the microscopic characteristics in the middle sampling site were investigated.

**FIGURE 4:**
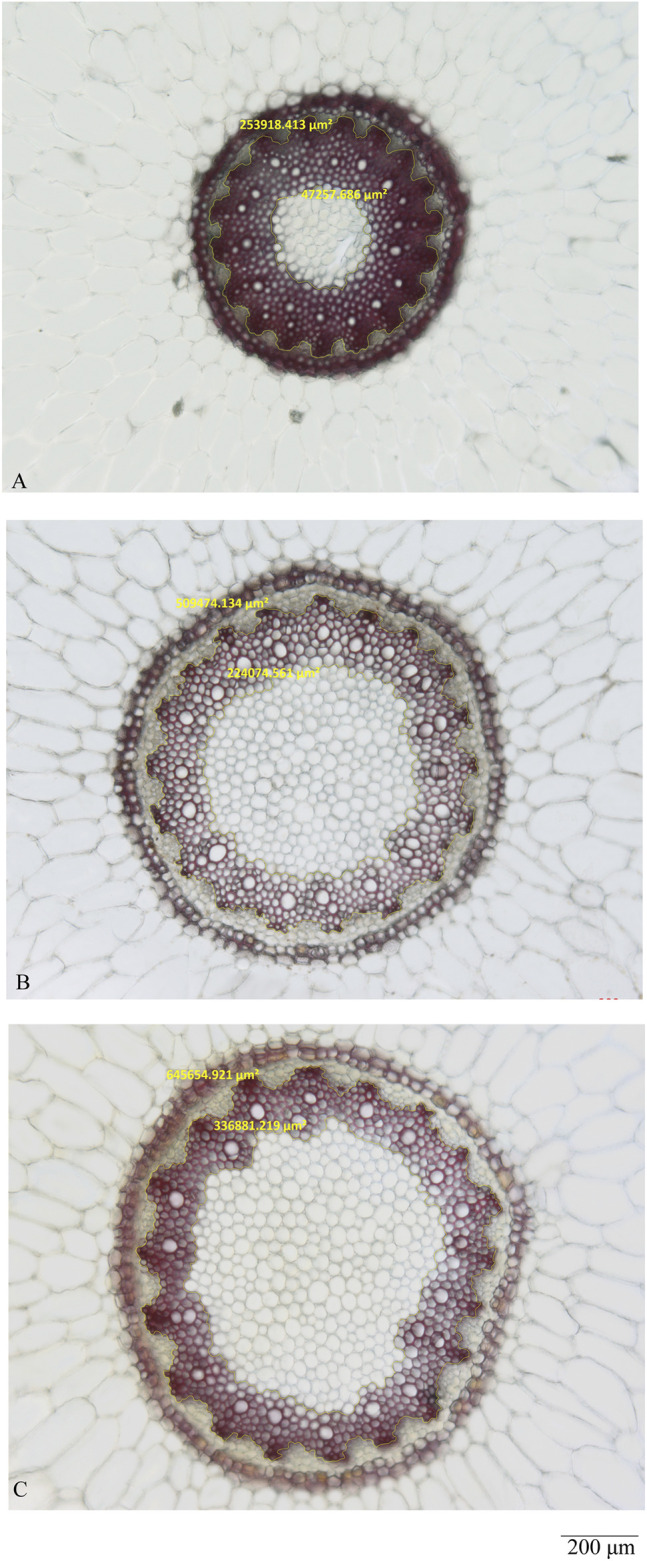
Measurement analysis by ZEN of the middle site: **(A)** Zhe-Maidong, **(B)** Chuan-Maidong, and **(C)** Chuan-Maidong with paclobutrazol.

As shown in Figures 3A–D (A-2, B-2, C-2, D-2), the pith and xylem area are clearly different among the three quality grades of Maidong. After staining with phloroglucinol test solution, only the xylem was stained red; thus, examining the boundary between xylem and pith/phloem was easy. The actual area of the selected scope was measured using the analysis function of ZEN software (Figure 4).

The pith and xylem areas in the middle sampling site of all samples were measured and calculated (Table 3). The pith area ranged from 24,675 to 188,958 μm^2^ for Zhe-Maidong, 140,699 to 383,775 μm^2^ for Chuan-Maidong, and 264,706 to 1,114,243 μm^2^ for Chuan-Maidong with paclobutrazol. The xylem area ranged from 152,195 to 446,337 μm^2^ for Zhe-Maidong, from 232,651 to 446,472 μm^2^ for Chuan-Maidong, and from 214,702 to 714,064 μm^2^ for Chuan-Maidong with paclobutrazol. The range of the area ratio between xylem and pith was as follows: 2.00–7.72 for Zhe-Maidong, 0.97–1.65 for Chuan-Maidong, and 0.40–0.96 for Chuan-Maidong with paclobutrazol.

**TABLE 3:** Measurement data of the three quality grades of Maidong in the middle sampling site.

Sample no.	Ⅰ^a^	Ⅱ^b^	Ⅲ^c^	Ⅲ/Ⅰ^d^	Sample no.	Ⅰ	Ⅱ	Ⅲ	Ⅲ/Ⅰ
Z1-1	81,090	358,251	277,161	3.42	CP2-1	469,260	814,575	345,315	0.74
Z1-2	47,258	253,918	206,660	4.37	CP2-2	338,453	642,992	304,539	0.90
Z1-3	36,115	250,914	214,799	5.95	CP2-3	388,940	662,765	273,825	0.70
Z1-4	188,958	635,295	446,337	2.36	CP2-4	518,334	858,267	339,933	0.66
Z2-1	170,782	512,002	341,220	2.00	CP3-1	604,054	961,707	357,653	0.59
Z2-2	114,201	454,009	339,808	2.98	CP3-2	673,572	1,160,622	487,050	0.72
Z2-3	116,845	380,068	263,223	2.25	CP3-3	626,398	993,179	366,781	0.59
Z2-4	24,675	215,227	190,552	7.72	CP3-4	917,850	1,364,305	446,455	0.49
Z3-1	157,485	470,416	312,931	2.00	CP4-1	870,824	1,253,498	382,674	0.44
Z3-2	29,963	182,158	152,195	5.08	CP4-2	636,175	958,408	322,233	0.51
Z3-3	96,574	376,058	279,484	2.89	CP4-3	540,641	865,545	324,904	0.60
Z3-4	107,117	392,831	285,714	2.67	CP4-4	293,372	559,209	265,837	0.91
Z4-1	145,391	476,613	331,222	2.28	CP5-1	515,175	958,198	443,023	0.86
Z4-2	126,981	382,109	255,128	2.00	CP5-2	341,307	592,817	251,510	0.74
Z4-3	165,293	496,785	331,492	2.00	CP5-3	494,032	857,986	363,954	0.74
Z4-4	115,890	453,956	338,066	2.92	CP5-4	264,706	499,548	234,842	0.89
C1-1	224,075	509,474	285,399	1.27	CP6-1	443,379	799,688	356,309	0.80
C1-2	255,424	534,656	279,232	1.10	CP6-2	408,180	743,671	335,491	0.82
C1-3	227,877	526,606	298,729	1.31	CP6-3	280,642	550,861	270,219	0.96
C1-4	140,699	373,350	232,651	1.65	CP6-4	679,345	979,165	299,820	0.44
C2-1	285,660	634,466	348,806	1.22	CP7-1	471,043	829,813	358,770	0.76
C2-2	374,893	799,515	424,622	1.13	CP7-2	437,266	785,005	347,739	0.80
C2-3	383,775	830,247	446,472	1.16	CP7-3	592,120	970,300	378,180	0.64
C2-4	334,601	660,323	325,722	0.97	CP7-4	336,881	645,655	308,774	0.92
C3-1	230,592	571,813	341,221	1.48	CP8-1	298,457	586,455	287,998	0.96
C3-2	210,726	522,545	311,819	1.48	CP8-2	363,258	654,104	290,846	0.80
C3-3	368,176	802,156	433,980	1.18	CP8-3	785,879	1,155,160	369,281	0.47
C3-4	299,166	659,963	360,797	1.21	CP8-4	721,453	1,012,287	290,834	0.40
C4-1	197,351	460,312	262,961	1.33	CP9-1	769,307	1,174,854	405,547	0.53
C4-2	210,724	490,761	280,037	1.33	CP9-2	746,678	1,166,125	419,447	0.56
C4-3	160,184	421,191	261,007	1.63	CP9-3	978,576	1,374,474	395,898	0.40
C4-4	258,882	532,082	273,200	1.06	CP9-4	1,114,243	1,606,081	491,838	0.44
CP1-1	583,132	1,005,278	422,146	0.72	CP10-1	465,625	802,405	336,780	0.72
CP1-2	951,800	1,665,864	714,064	0.75	CP10-2	525,828	933,723	407,895	0.78
CP1-3	329,114	543,816	214,702	0.65	CP10-3	387,022	674,291	287,269	0.74
CP1-4	450,713	750,615	299,902	0.66	CP10-4	320,777	584,877	264,100	0.82

^a^ The area of pith.

^b^ The area of xylem plus pith.

^c^ The area of xylem (*Ⅱ* minus *Ⅰ*).

^d^ The area ratio between xylem and pith.

**FIGURE 5:**
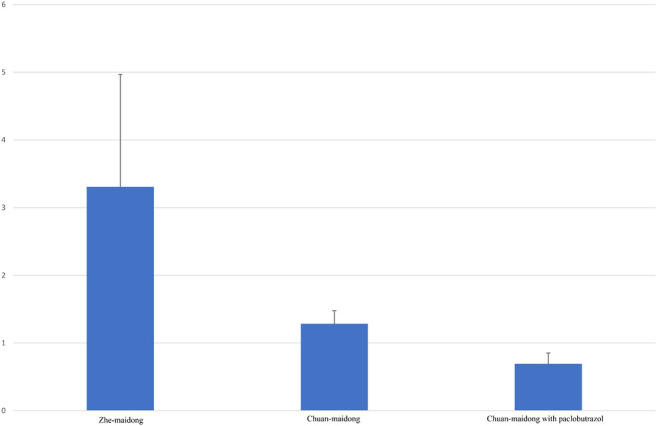
Area ratio between xylem and pith in different quality grades of Maidong. The values are mean ± SD, n = 16 in Zhe-Maidong and Chuan-Maidong and n = 40 in Chuan-Maidong with paclobutrazol.

After factoring in the area ratio of each sample, the mean and standard deviation (SD) of the three grades of Maidong were calculated (Table 4). A chart was generated to show the relationship of the area ratio between xylem with the pith and three quality grades of Maidong. According to these numeric values, the chart indicates a method by which the three grades of Maidong could be quickly and efficiently distinguished by the area ratio between xylem and pith (Figure 5).

**TABLE 4:** Mean and SD of the area ratio between xylem and pith of each grades.

Maidong	Area ratio between xylem and pith
Zhe-Maidong	3.306 ± 1.663 (mean ± SD, n = 16)
Chuan-Maidong	1.282 ± 0.196 (mean ± SD, n = 16)
Chuan-Maidong with paclobutrazol	0.691 ± 0.161 (mean ± SD, n = 40)

Variance analysis of the area ratio between xylem and pith of the three quality grades of Maidong was performed with SPSS (F = 62.842, *p* < 0.001). Results show a highly significant difference between Zhe-Maidong and Chuan-Maidong and Zhe-Maidong and Chuan-Maidong with paclobutrazol (both *p* < 0.001) and a significant difference between Chuan-Maidong and Chuan-Maidong with paclobutrazol (*p* = 0.041).

### Evaluation Maidong in Herbal Markets and Botanical Pharmacies

Twelve batches of Maidong obtained from Chinese herbal markets and botanical pharmacies were analyzed. All samples were crude drugs without cultivation information. Results showed that the area ratio between xylem and pith of all samples ranged 0.41–1.06 (Table 5).

**TABLE 5:** Measurement data of Maidong from the herbal markets and botanical pharmacies.

Sample no.	Ⅰ^a^	Ⅱ^b^	Ⅲ^c^	Ⅲ/Ⅰ^d^	Sample no.	Ⅰ	Ⅱ	Ⅲ	Ⅲ/Ⅰ
MS1-1	761,648	1,160,868	399,220	0.52	MS7-1	367,018	584,475	217,457	0.59
MS1-2	349,188	568,929	219,741	0.63	MS7-2	493,800	766,032	272,232	0.55
MS1-3	420,024	751,610	331,586	0.79	MS7-3	406,414	679,591	273,177	0.67
MS1-4	1,172,162	1,650,899	478,737	0.41	MS7-4	698,200	1,117,552	214,176	0.61
MS2-1	501,344	786,097	284,753	0.57	MS8-1	419,892	653,733	233,841	0.56
MS2-2	335,461	595,213	259,752	0.77	MS8-2	431,640	764,408	332,768	0.77
MS2-3	497,161	826,628	329,467	0.66	MS8-3	714,762	1,349,919	635,157	0.89
MS2-4	408,744	695,316	286,572	0.70	MS8-4	424,400	741,024	316,624	0.75
MS3-1	852,114	1,300,519	448,405	0.53	MS9-1	660,896	1,052,320	391,424	0.59
MS3-2	364,316	623,910	259,594	0.71	MS9-2	510,832	774,384	263,552	0.52
MS3-3	341,386	556,751	215,365	0.63	MS9-3	482,832	712,928	230,096	0.48
MS3-4	335,953	644,354	308,401	0.92	MS9-4	342,560	634,768	292,208	0.85
MS4-1	272,036	493,928	221,892	0.82	MS10-1	834,652	1,360,482	525,830	0.63
MS4-2	568,621	948,040	379,419	0.67	MS10-2	675,437	1,154,997	479,560	0.71
MS4-3	488,984	728,193	239,209	0.49	MS10-3	413,458	748,358	334,900	0.81
MS4-4	333,198	630,627	297,429	0.89	MS10-4	365,905	684,242	318,337	0.87
MS5-1	456,148	743,759	287,611	0.63	MS11-1	254,019	497,877	243,858	0.96
MS5-2	435,293	679,153	243,860	0.56	MS11-2	453,317	788,771	335,454	0.74
MS5-3	313,637	555,578	241,941	0.77	MS11-3	543,809	919,037	375,228	0.69
MS5-4	147,068	304,076	157,008	1.07	MS11-4	379,567	713,586	334,018	0.88
MS6-1	258,416	490,179	231,763	0.90	MS12-1	756,490	1,202,819	446,329	0.59
MS6-2	365,085	592,346	227,261	0.62	MS12-2	694,456	1,159,741	465,285	0.67
MS6-3	430,287	687,642	257,355	0.60	MS12-3	574,239	970,463	396,224	0.69
MS6-4	244,198	479,189	234,991	0.96	MS12-4	338,910	647,318	308,408	0.91

^a^ The area of pith.

^b^ The area of xylem plus pith.

^c^ The area of xylem (*Ⅱ* minus *Ⅰ*).

^d^ The area ratio between xylem and pith.

Moreover, the area ratio between xylem and pith was pairwise compared between Maidong in the market and three grades of Maidong with SPSS: Maidong in the market vs. Zhe-Maidong (*p* < 0.001), Maidong in the market vs. Chuan-Maidong (*p* < 0.001), and Maidong in the market vs. Chuan-Maidong with paclobutrazol (*p* = 0.915). Data of the market samples were not significantly different from those of Chuan-Maidong with paclobutrazol. Therefore, it was deduced that all the samples from the market were Chuan-Maidong with paclobutrazol.

### Method Validation by LC/MS

**FIGURE 6:**
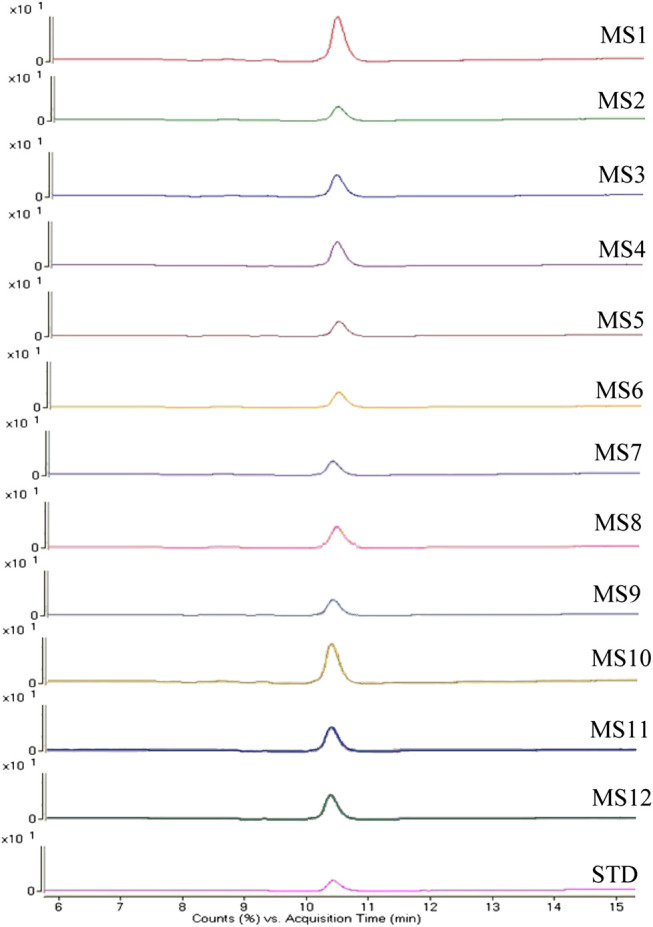
Mass spectrometry (MS) spectra of paclobutrazol in herbal markets and botanical pharmacies samples and standard substance.

To validate the accuracy of the method to distinguish the three quality grades of Maidong based on microscopic quantification, the market samples were tested by LC/MS. Qualitative analysis of paclobutrazol was conducted using the multiple reaction monitoring mode, in which monitoring of precursor ion to product ion transitions of m/z 294 → m/z 70 and m/z 125 for paclobutrazol was performed. The MS spectra of the analytes are shown in Figure 6. Results revealed that the twelve batches of Maidong from the herbal markets and botanical pharmacies had the same ion chromatograms as those of paclobutrazol standard solutions, thereby confirming that Maidong samples from the market were Chuan-Maidong with paclobutrazol. The LC/MS findings proved the accuracy of microscopic quantification in identifying the quality grades of Maidong.

### Method Extension

This study established an analytical method to identify and evaluate three quality grades of Maidong using the ZEISS microscope and ZEN software. The critical quantification information is the area ratio between xylem and pith. The ratio is a relative rather than an absolute value.

In theory, if image software could indicate information such as pixels of a selected area, then the pixel ratio should be equal to the area ratio and thus could be used to identify three quality grades of Maidong.

**FIGURE 7:**
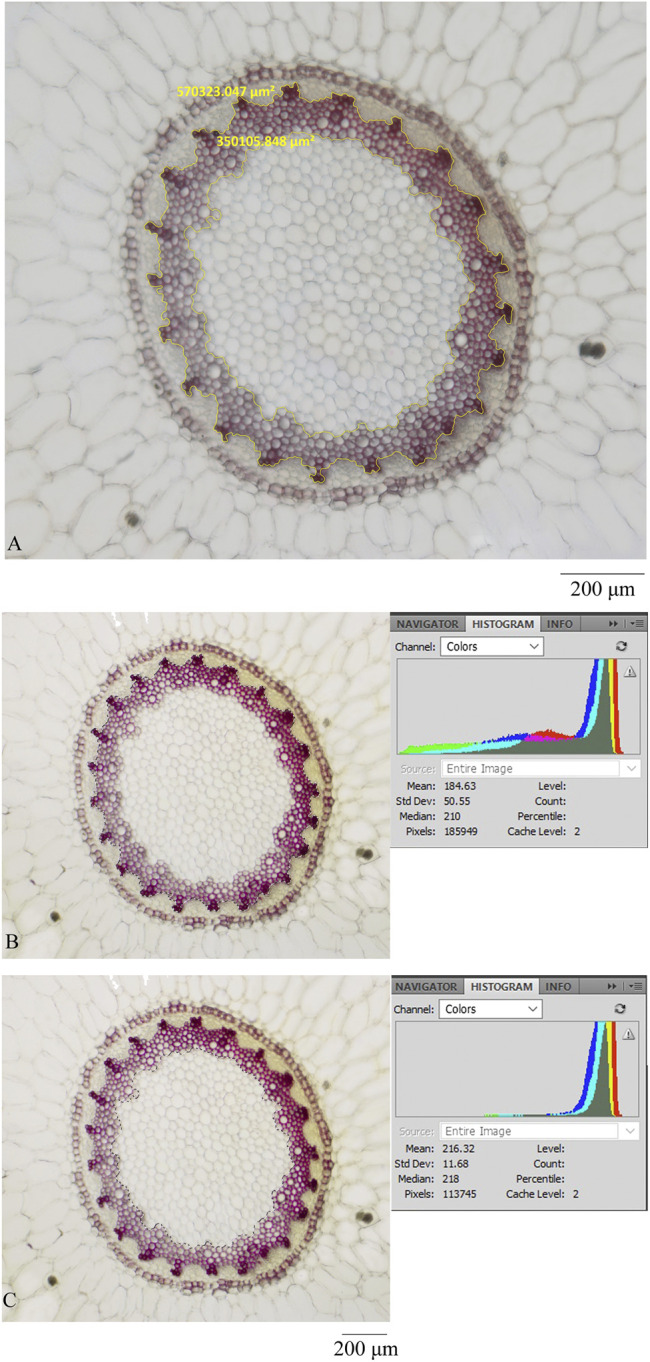
Transverse section was processed by different microscopes and software: **(A)** ZEISS and ZEN, **(B)** OLYMPUS and photoshop (showing the pixel of xylem and pith), and **(C)** OLYMPUS and photoshop (showing the pixel of pith).

To verify this assumption, this study selected a section of Maidong randomly from market samples and acquired an image of the stele using an OLYMPUS microscope; thereafter, the image was analyzed this picture with the commonly used image processing software Photoshop CS2 (Adobe, CA). The pith and xylem areas were selected separately using a polygonal lasso tool. The pixel of the selected area was shown in a histogram, and the pixel ratio between xylem and pith was calculated. Results showed that the pixel ratio between xylem and pith was equal to the area ratio between xylem and pith (Figure 7, Table 6).

**TABLE 6:** Measurement data by different microscopes and software.

Microscope+software	Pith	Pith+xylem	Xylem	Xylem/pith
ZEISS+ZEN	350,106 μm^2^	570,323 μm^2^	220,218 μm^2^	0.63
OLYMPUS+photoshop	113,745 pixel	185,949 pixel	73,133 pixel	0.63

The quantification data are based on a relative value; thus, the method could be used to distinguish three grades of Maidong without the constraints associated with the type of themicroscope and processing software.

## Discussion

In this study, we evaluated the microscopic characteristics of Maidong from three quality grades; the result showed the pith and xylem area in the middle sampling site, the thickness and lignification of endodermis and stone cells, and the shape of the protoxylem and lignified degree in the lower sampling site different among the three quality grades of Maidong. Our findings reveal that cultivation age, area, and especially plant growth regulator could influence the anatomical characteristics of Maidong. The area ratio between xylem and pith, which is an objective and practical microscopic characteristic, was first developed to distinguish the different quality grades of Maidong; the area ratio varied according to the quality grades as follows: Zhe-Maidong, 3.306 ± 1.663 (mean ± SD, n = 16); Chuan-Maidong, 1.282 ± 0.196 (mean ± SD, n = 16); and Chuan-Maidong with paclobutrazol, 0.691 ± 0.161 (mean ± SD, n = 40). Consequently, a novel microscopic quantification method to distinguish the three quality grades of Maidong was established.

The identification of three quality grades of Ophiopogonis Radix will make efforts on three aspects: the recovery of superior germplasm resource of Zhe-Maidong, provide evidence to customers and herbalists who want better quality of Maidong, and the improvement quality of Maidong in market and botanical pharmacies. Our study is the first to apply microscopic techniques to test and distinguish Ophiopogonis Radix from different regions and with different cultivation modes simultaneously. It is also the first to use that microscopic quantitative method to classify the quality grades of herbal medicine. This research largely expanded the application range of microscopic technology and provided another way to evaluate the quality grades of herbal medicine.

Microscopic quantification avoids subjective and relative factors in microscope research. Generally, quantification of microscopic characteristics is influenced by sampling site; thus, in most cases, the diameter of the sampling site should be restricted in microscopic quantification analysis, which limits the practical application of this method. For example, the identification of Wuzhimaotao (Radix Fici Hirtae) by quantification used samples around 1 cm in diameter (Au et al., 2009). In our research, the sampling site was considered and investigated. Results showed that the microscopic quantification of the middle sampling site of three quality grades of Maidong is stable, consistent and subject to quantification, and reveals important distinctions. Maidong is a fusiform tuber, and the middle location is easily determined; hence, there is no need to restrict the diameter of sampling site. The practicability of this method could be improved.

Currently, not all microscopes equipped with software can measure actual areas; thus, measurement of actual areas is limited to several types of microscopes and software. In this study, the identification characteristic is based on a ratio, which is a relative value. In theory and in practice, microscopic images from any type of the microscope could be processed using normal image processing software to obtain the pixel value of selected areas. The pixel ratio is equal to the area ratio, which means that our novel method is not limited by the type of the microscope and software, thereby further expanding the scope of application.

For Chuan-Maidong with paclobutrazol, the microscopic characteristics of lower sampling site were not stable, and almost half of samples have undeveloped metaxylem. We assumed that was resulted of short cultivation age and the influence of plant growth regulator. We will confirm this hypothesis in the future studies.

In conclusion, we established an efficient, convenient, and practical method evaluating the quality grades of Maidong based on microscopic quantification, which could in turn improve the quality and safety of Maidong in China, specifically. Furthermore, our study also expands the application of quantitative microscopic techniques and provides another way to identify the quality grades of herbal medicine.

## Data Availability Statement

The raw data supporting the conclusions of this article will be made available by the authors, without undue reservation.

## Author Contributions

KY conceived and designed the study, did literature research, and prepared the manuscript; WL collected LC-MS data; NZ did the statistical analysis; XC did the data acquisition; SZ collected the plant material; TZ edited the manuscript; SK identified the plant material; FW and SM guaranteed integrity of the entire study and reviewed the manuscript.

## Conflict of Interest

The authors declare that the research was conducted in the absence of any commercial or financial relationships that could be construed as a potential conflict of interest.
